# Clinical Implication of Systemic Immune-Inflammation Index and Prognostic Nutritional Index in Skull Base Chordoma Patients

**DOI:** 10.3389/fonc.2021.548325

**Published:** 2021-02-25

**Authors:** Mingxuan Li, Jiwei Bai, Shuai Wang, Yixuan Zhai, Shuheng Zhang, Chuzhong Li, Jiang Du, Yazhuo Zhang

**Affiliations:** ^1^Beijing Neurosurgical Institute, Capital Medical University, Beijing, China; ^2^Department of Neurosurgery, Beijing Tiantan Hospital, Capital Medical University, Beijing, China; ^3^Department of Neurosurgery, The First Affiliated Hospital of Zhengzhou University, Zhengzhou, China; ^4^Department of Neuropathology, Beijing Neurosurgical Institute, Capital Medical University, Beijing, China; ^5^Beijing Institute for Brain Disorders Brain Tumor Center, Beijing, China; ^6^China National Clinical Research Center for Neurological Diseases, Beijing, China; ^7^Key Laboratory of Central Nervous System Injury Research, Capital Medical University, Beijing, China

**Keywords:** skull base chordoma, systemic immune-inflammation index, prognostic nutritional index, overall survival, prognosis

## Abstract

Inflammation associated markers and nutritional indexes are associated with survival, and act as novel prognostic grading systems in patients with cancer, though the role of these markers in chordoma remains unclear. The current study aimed to characterize systemic immune-inflammation index (SII) and prognostic nutritional index (PNI), and their relationship with clinicopathological data and survival in skull base chordoma. Our retrospective study enrolled 183 patients with primary skull base chordoma who received surgical treatment. Clinicopathological data and preoperative blood tests including neutrophil, lymphocyte, platelet counts and albumin level were collected from medical records. Neutrophil lymphocyte ratio (NLR), platelet lymphocyte ratio (PLR), SII, PNI were calculated and the optimal cut-off values of these markers were used for further survival analysis via Kaplan–Meier survival analysis and Cox proportional hazards regression analysis. The value of NLR, PLR, SII, and PNI in skull base chordoma ranged from 0.44–6.48, 45.36–273.94, 113.37–1761.45, and 43.40–70.65, respectively. PNI was significantly correlated with patients' sex (*p* = 0.005) and age (*p* = 0.037). SII was positively correlated with NLR and PLR, but negatively correlated with PNI. The median overall survival (OS) time was 74.0 months and Kaplan–Meier survival analysis indicated that all four indexes were associated with OS. Multivariable Cox proportional hazards regression analysis identified that high SII was an independent prognostic factor for poor OS. More importantly, patients with high SII and PNI had the worst outcomes and combined use of SII and PNI increased the predictive ability for patients' survival in skull base chordoma. Our results suggest SII and PNI may be effective prognostic indicators of OS for patients with primary skull base chordoma after surgical resection.

## Introduction

Chordoma is a comparatively rare, aggressive, slow-growing tumor originating from notochord remnants (1, 2). Chordoma largely occurs at the axial skeleton, preferentially in the skull base and sacrococcygeal region (1, 3). Radical surgical resection and postoperative radiotherapy have been recognized to increase the opportunity of favorable survival in chordoma patients (4, 5). However, the recurrence rate is high even patients receive radical surgery and radiotherapy, resulting in a dismal prognosis. Recent studies have indicated that targeted therapeutics, including afatinib and imatinib, show potential therapeutic value in clinical trials, though the benefit remains limited (6, 7). Moreover, clinical prognostic grading systems for chordoma patients remain unsatisfactory. Thus, better understanding treatment strategies and the need for possible prognostic grading systems to identify patients with high risk of death in chordoma is needed.

There is abundant evidence showing that immune cells such as neutrophils, monocytes, lymphocytes, and platelets, play an essential part in the tumor development, resistance to therapy and outcome of cancer patients (8–11). Inflammatory associated markers consisting of neutrophil lymphocyte ratio (NLR) and platelet lymphocyte ratio (PLR) exhibit powerful prognostic value in various solid tumors, such as hepatocellular carcinoma (12, 13), breast tumors (14), and malignant esophageal tumors (15). More recently, systemic immune-inflammation index, simplified as SII, an integrated analysis of neutrophil, platelet and lymphocyte, was developed and recognized as a novel independent indicator of survival in several malignancies (16, 17).

Prognostic nutritional index (PNI), an evaluation index indicating immunological and nutritional status in patients defined as serum albumin plus lymphocyte count, has been considered of unique prognostic values in various cancers (18–20). Recently, Hu et al. (21) reported the potential prognostic role of several inflammatory biomarkers in chordoma patients, however, PNI were not included and study aiming to explore the prognostic effect of nutrition and inflammation in chordoma patients remain lacking. Thus, in the present study, we characterized preoperative inflammatory biomarkers including NLR, PLR, SII, and PNI. We then evaluated the association of these measures to clinicopathologic data and outcome in patients with primary skull base chordoma who underwent surgical resection. Finally, we evaluated the correlation of PNI with other markers, and compared the prognostic performance of those indexes in skull base chordoma.

## Materials and Methods

### Patients Selection

One hundred and eighty-three patients diagnosed with clival chordoma who received surgical resection in Beijing Tiantan Hospital from January 2008 to September 2014, were enrolled in the current retrospective research. The inclusive criteria for this study were: (1) pathology confirmed chordoma tumor located at the skull base; (2) primary tumor with no prior history of surgical therapy, radiotherapy, or chemotherapy; and (3) availability of complete clinical information, preoperative blood laboratory information, and follow up data. Patients with preoperative therapy, proof indicating acute or chronic inflammatory disease, incomplete clinicopathological information or laboratory data were excluded.

### Information Collection

Clinical data including patients' age, sex, tumor volume, tumor texture (soft, tumors can be easily suctioned with an aspirator; hard, tumors can hardly be removed without the help of scissor or punch forceps; moderate, between soft and hard type), tumor blood supply (rich, tumor resection surface is liable to bleed and hard to aspirate clearly; poor, tumors with limited bleeding and is easy to aspirate clearly; moderate, between poor and rich supply) (22), tumor pathology (classical, chondroid, and dedifferentiated chordoma) (1), brainstem involvement (yes or no, acquired from preoperative images), Al-Mefty classification (type I, II, and III), degree of resection (total resection: no remaining tumor based on pre-operative and post-operative image examinations; subtotal resection: resection percent ≥95% and part resection <95%) (23) and pre-operative blood tests for neutrophil (10^9^/L), lymphocyte (10^9^/L), platelet counts (10^9^/L), and albumin level (g/L) were collected from medical record system. Inflammatory associated markers were measured as followed in current study (19): NLR = neutrophil/lymphocyte; PLR = platelet/lymphocyte; SII = platelet × neutrophil/lymphocyte and PNI = albumin level + 5 × lymphocyte.

### Follow Up

Patients were followed up by regular image examination and clinical evaluation till October 2019 via outpatient examination and telephone interview. Overall survival (OS) was defined as the interval between tumor resection to death. For patients who were alive until October 2019, observations were censored.

### Cutoff Values for NLR, PLR, SII, and PNI

We used X-tile software (Version 3.61, Yale University, New Haven, CT, USA), a tool to discover outcome-based cutoff value, to evaluate the ideal cut-off values of NLR, PLR, SII, and PNI (16). The optimal cutoff values were further recognized as the values with the lowest *p*-value via the log-rank test in overall survival analysis.

### Statistical Analysis

Statistical analyses were performed using IBM SPSS Statistics for Windows, version 19.0 (IBM Corp., Armonk, NY, USA) and GraphPad Prism (Version 7.0, GraphPad, La Jolla, CA, USA). Differences between categorical factors were determined by chi-squared test. Correlations between inflammatory markers were analyzed using Pearson correlation. Kaplan–Meier survival method was used for survival analysis, and the log-rank test was used to find differences between groups. Cox proportional regression hazard models were used to identify potential independent prognostic variables, and factors significant in univariable analysis were enrolled in the multivariable analysis. The bootstrap resample analysis with 1,000 repeats (24), which was widely used to test the reproducibility of survival data via randomly sampling patients from the same cohort, was performed to validate our results (25–27). Receiver operating characteristic (ROC) curve analysis was applied to evaluate the prognostic capacity of these inflammatory markers for 3-year and 5-year OS. All tests were 2-sided, and a *p-*value <0.05 was considered of statistically significance.

## Results

### Patients Summary

Patient descriptive characteristics are reported in Table 1. Between January 2008 and September 2014, 183 patients (96 males and 87 females), with a median age of 41 years, enrolled in the current study. Based on tumor pathology classified as classical, chondroid and dedifferentiated chordoma (1), 125 patients were diagnosed as classical chordoma and 58 patients had chondroid chordoma. No dedifferentiated type was observed. The tumor volume ranged from 1740.4 to 258025.6 mm^3^ (median 21000.0 mm^3^). For tumor texture, 55 patients were examined to have a soft tumor, while 128 patients harbored hard or moderate tumors. Tumor blood supply was rich in 107 patients and poor to moderate blood supply was observed in 76 patients. Brainstem involvement was observed in 115 patients. Thirty three patients had Al-Mefty type I tumors, 87 patients harbored Al-Mefty type II tumors, and 63 patients had Al-Mefty type III tumors. Seventy one patients received postoperative radiotherapy, and most of them (41 patients) received gamma knife therapy.

**Table 1:** Association between the NLR, PLR, SII, PNI, and clinicopathological features of skull base chordoma patients.

**Variables**		**NLR**			**PLR**			**SII**			**PNI**		
	**N**	**High**	**Low**	***P-*value**	**High**	**Low**	***P-*value**	**High**	**Low**	***P-*value**	**High**	**Low**	***P-*value**
Total	183	79	104		51	132		46	137		110	73	
Sex				0.666			0.010^*^			0.700			0.005^*^
Male	96	40	56		19	77		23	73		67	29	
Female	87	39	48		32	55		23	64		43	44	
Age, years				0.390			0.639			0.668			0.037^*^
≤55	151	63	88		41	110		37	114		96	55	
>55	32	16	16		10	22		9	23		14	18	
Tumor volume, mm^3^				0.470			0.025^*^			0.067			0.879
≤20,000	89	36	53		18	71		17	72		54	35	
>20,000	94	43	51		33	61		29	65		56	38	
Texture				0.002^*^			0.633			0.155			0.498
Soft	55	14	41		14	41		10	45		31	24	
Others (hard or moderate)	128	65	63		37	91		36	92		79	49	
Blood supply				0.249			0.784			0.078			0.259
Rich	107	50	57		29	78		32	75		68	39	
Others (poor or moderate)	76	29	47		22	54		14	62		42	34	
Pathology				0.204			0.954			0.603			0.965
Classical	125	50	75		35	90		30	95		75	50	
Chondroid	58	29	29		16	42		16	42		35	23	
Brainstem involvement				0.300			0.506			0.072			0.969
No	68	26	42		17	51		12	56		41	27	
Yes	115	53	62		34	81		34	81		69	46	
Al-Mefty classification				0.812			0.360			0.083			0.893
Type I	33	13	20		6	27		6	27		21	12	
Type II	87	37	50		25	62		18	69		52	35	
Type III	63	29	34		20	43		22	41		37	26	
Postoperative radiotherapy	71												
Gamma knife	41												
Proton beam therapy	8												
Carbon ion therapy	1												
Cyberknife	1												
Intensity-modulated therapy	4												
Unclear	16												

* *p < 0.05; NLR, neutrophil lymphocyte ratio; PLR, platelet lymphocyte ratio; SII, systemic immune-inflammation index; PNI, prognostic nutritional index*.

### Identify of the Optimal Cutoff Values for Inflammation-Based Markers

The NLR, PLR, SII, and PNI values in skull base chordoma ranged from 0.44–6.48, 45.36–273.94, 113.37–1761.45, and 43.40-70.65, respectively. To assess the best cut-off value, X-tile software was applied and the best cut-off values for NLR, PLR, SII, PNI were 2.10, 152.40, 600.50, 54.55, respectively, in the OS analysis (Supplementary Figure 1). Consequently, patients were separated into two groups: high (≥2.10, *n* = 79) or low (<2.10, *n* = 104) NLR group; high (≥152.40, *n* = 51) or low (<152.4, *n* = 132) PLR group; high SII (≥600.50, *n* = 46) or low (<600.50, *n* = 137) SII group; high PNI (≥54.55, *n* = 110) or low (<54.55, *n* = 73) PNI group for further analysis.

### Association Between NLR, PLR, SII, PNI, and Clinical Parameters

We next analyzed the correlation between inflammation based biomarkers and clinical features in chordoma patients. Our analysis showed that high NLR was correlated with hard or moderate texture (*p* = 0.002), we did not observe any significant associations between NLR with sex, age, tumor volume, blood supply, pathology, or brainstem involvement. For PLR, there were 32 (32/51) females in the high PLR group and 55 (55/132) females in the other group (*p* = 0.010). Moreover, high PLR was significantly related with bigger tumor volume (*p* = 0.025) compared with low PLR.

Patients with high SII trended to have rich tumor blood supply (*p* = 0.078), bigger tumor volume (*p* = 0.067), Al-Mefty classification type III (*p* = 0.083), and brainstem involvement (*p* = 0.072) when compared with chordoma patients in low SII group, though these findings were not significant (all *p-*values >0.05). When considering PNI, our results showed that low PNI was correlated with older age (*p* = 0.005) and female gender (*p* = 0.037) compared to those with a high PNI. No other significant associations were observed in current study (Table 1).

We also analyzed the correlation between inflammatory base biomarkers, and found a significantly positive correlation between SII and NLR or PLR. Furthermore, PNI was negatively correlated with the other three markers (*r* = −0.459, −0.571, −0.345 for NLR, PLR, SII, respectively, *p* < 0.001).

### Survival Analysis

**Figure 1:**
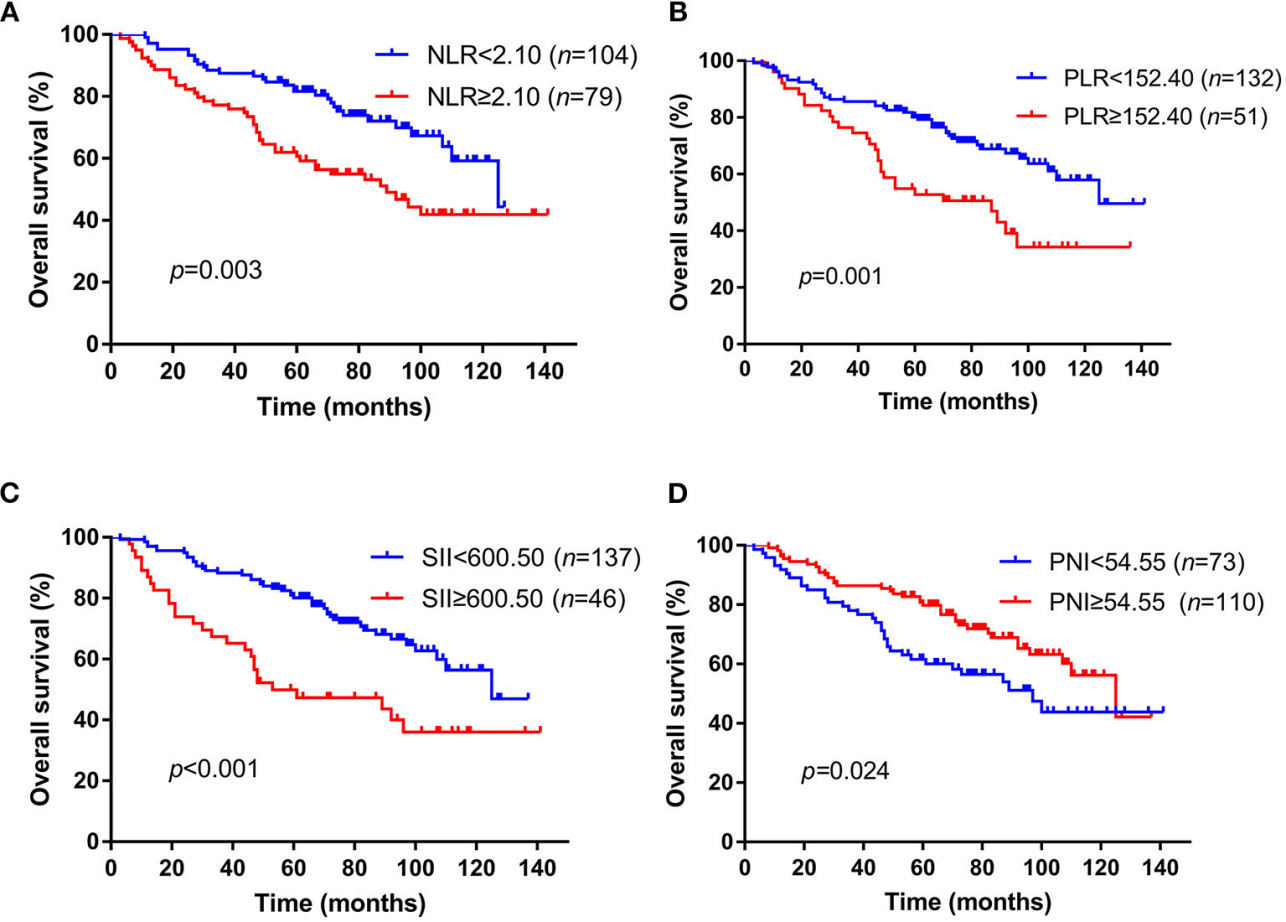
Kaplan–Meier survival curves of NLR, PLR, SII, and PNI for OS in skull base chordoma patients. **(A)** Patients with high NLR had worse OS than those with low NLR (*p* = 0.003). **(B)** Patients with high PLR had worse OS than those with low PLR (*p* = 0.001). **(C)** Patients with high SII had worse OS than those with low SII (*p* < 0.001). **(D)** Patients with low PNI had worse OS than those with high PNI (*p* = 0.024). NLR, neutrophil lymphocyte ratio; PLR, platelet lymphocyte ratio; SII, systemic immune-inflammation index; PNI, prognostic nutritional index; OS, overall survival.

The median follow-up time was 74 months (range, 3–141 months), 128 patients got recurrence and 72 patients died during the time of follow up in this cohort. The median OS time was 74.0 months with a 3-year OS rate of 83.1% and 5-year OS rate of 67.8%. We then used the Kaplan–Meier method to assess the correlation between these inflammation markers and patients' OS. Patients NLR ≥2.10 had a significantly worse OS (median time: 89 months) than patients with NLR <2.10 (median OS: 125 months, *p* = 0.003) (Figure 1A). For PLR, the median OS time was 87 months in patients with PLR ≥152.40, which was significantly shorter than the median OS time (125 months) in the other group (*p* = 0.001) (Figure 1B). For SII and PNI, patients with an SII ≥600.50 was associated with a notably shorter OS time than those with SII <600.50 (median OS: 53 and 125 months, respectively, *p* < 0.001) (Figure 1C), while patients with low PNI had a worse survival compared to patients with high PNI (median OS: 97 and 125 months, respectively, *p* = 0.024) (Figure 1D). The bootstrap analysis further revealed the reliability of the prognostic role of NLR, PLR, SII, and PNI (Supplementary Figure 2).

**Figure 2:**
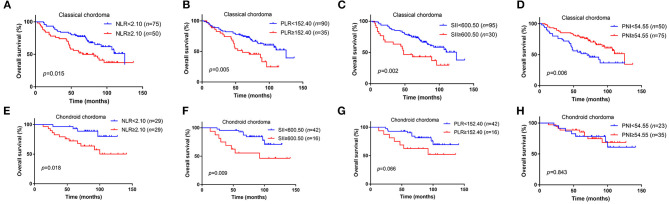
Kaplan–Meier survival curves of NLR, PLR, SII, and PNI for OS in different pathological types of chordoma. **(A)** High NLR was associated with poor OS in classical chordoma (*p* = 0.015). **(B)** High PLR was associated with poor OS in classical chordoma (*p* = 0.005). **(C)** High SII was associated with poor OS in classical chordoma (*p* = 0.002). **(D)** Low PNI was associated with poor OS in classical chordoma (*p* = 0.006). **(E)** High NLR was associated with poor OS in chondroid chordoma (*p* = 0.018). **(F)** High SII was associated with poor OS in chondroid chordoma (*p* = 0.009). **(G)** No significant association was observed between PLR and OS in chondroid chordoma (*p* = 0.066). **(H)** No significant association was observed between PNI and OS in chondroid chordoma (*p* = 0.843). NLR, neutrophil lymphocyte ratio; PLR, platelet lymphocyte ratio; SII, systemic immune-inflammation index; PNI, prognostic nutritional index; OS, overall survival.

We further analyzed the value of these markers in different pathological type of chordoma, and our results elucidated that high NLR (*p* = 0.015), high PLR (*p* = 0.005), high SII (*p* = 0.002), low PNI (*p* = 0.006) were associated with poor OS in classical chordoma (Figures 2A–D). For chondroid chordoma, NLR (*p* = 0.018) and SII (*p* = 0.009) rather than PLR (*p* = 0.066) or PNI (*p* = 0.843) were significantly correlated with patients' OS (Figures 2E–H).

In the univariable Cox analysis, age (*p* = 0.018), tumor volume (*p* = 0.028), blood supply (*p* = 0.014), pathology (*p* = 0.022), Al-Mefty classification (*p* = 0.008), degree of resection (*p* < 0.001), recurrence (*p* < 0.001), NLR (*p* = 0.003), PLR (*p* = 0.001), SII (*p* < 0.001), PNI (*p* = 0.026) were considered as factors affecting OS (Table 2). To identify possible independent factors, multivariable Cox proportional hazards regression analysis including these variables were preformed and the results indicated that degree of resection (*p* = 0.003), recurrence (*p* < 0.001), and SII (*p* = 0.001) were able to independently predict patient OS. It was worthy to note that PNI trended toward significant in the multivariable Cox analysis (*p* = 0.058) (Table 2).

**Table 2:** Univariable and multivariable cox analysis of overall survival in skull base chordoma.

**Variables**	**Univariable analysis**			**Multivariable analysis**		
	**HR**	**95% CI**	***P-*value**	**HR**	**95% CI**	***P-*value**
Sex (female/male)	0.981	0.617–1.561	0.937			
Age, years (>55/≤55)	1.935	1.120–3.343	0.018^*^	NA	NA	0.081
Tumor volume, mm^3^ (>20,000/≤20,000)	1.702	1.059–2.737	0.028^*^	NA	NA	0.593
Texture (hard or moderate/soft)	1.549	0.899–2.670	0.115			
Blood supply (poor or moderate/rich)	0.527	0.317–0.876	0.014^*^	NA	NA	0.408
Pathology (chondroid /classical)	0.523	0.300–0.912	0.022^*^	NA	NA	0.129
Brainstem involvement (yes/no)	1.038	0.645–1.669	0.878			
Al-Mefty classification (type III versus type I and II)	1.884	1.183–3.000	0.008	NA	NA	0.993
Degree of resection (part /subtotal or total resection)	3.048	1.913–4.854	<0.001^*^	2.076	1.292-3.335	0.003^*^
Postoperative radiotherapy (yes/no)	0.769	0.464–1.276	0.309			
Recurrence (yes/no)	9.192	3.352–25.205	<0.001^*^	7.144	2.565−19.901	<0.001^*^
NLR (high/low)	2.016	1.264–3.216	0.003^*^	NA	NA	0.152
PLR (high/low)	2.160	1.346–3.467	0.001^*^	NA	NA	0.094
SII (high/low)	2.375	1.471–3.835	<0.001^*^	2.205	1.363-3.568	0.001^*^
PNI (high/low)	0.592	0.372–0.940	0.026^*^	NA	NA	0.058

* *p < 0.05; NLR, neutrophil lymphocyte ratio; PLR, platelet lymphocyte ratio; SII, systemic immune-inflammation index; PNI, prognostic nutritional index; HR, hazard ratio; CI, confidence interval; NA, not acquired*.

### Comparison of the Predictive Value of NLR, PLR, SII, and PNI for 3-year and 5-year OS

**Figure 3:**
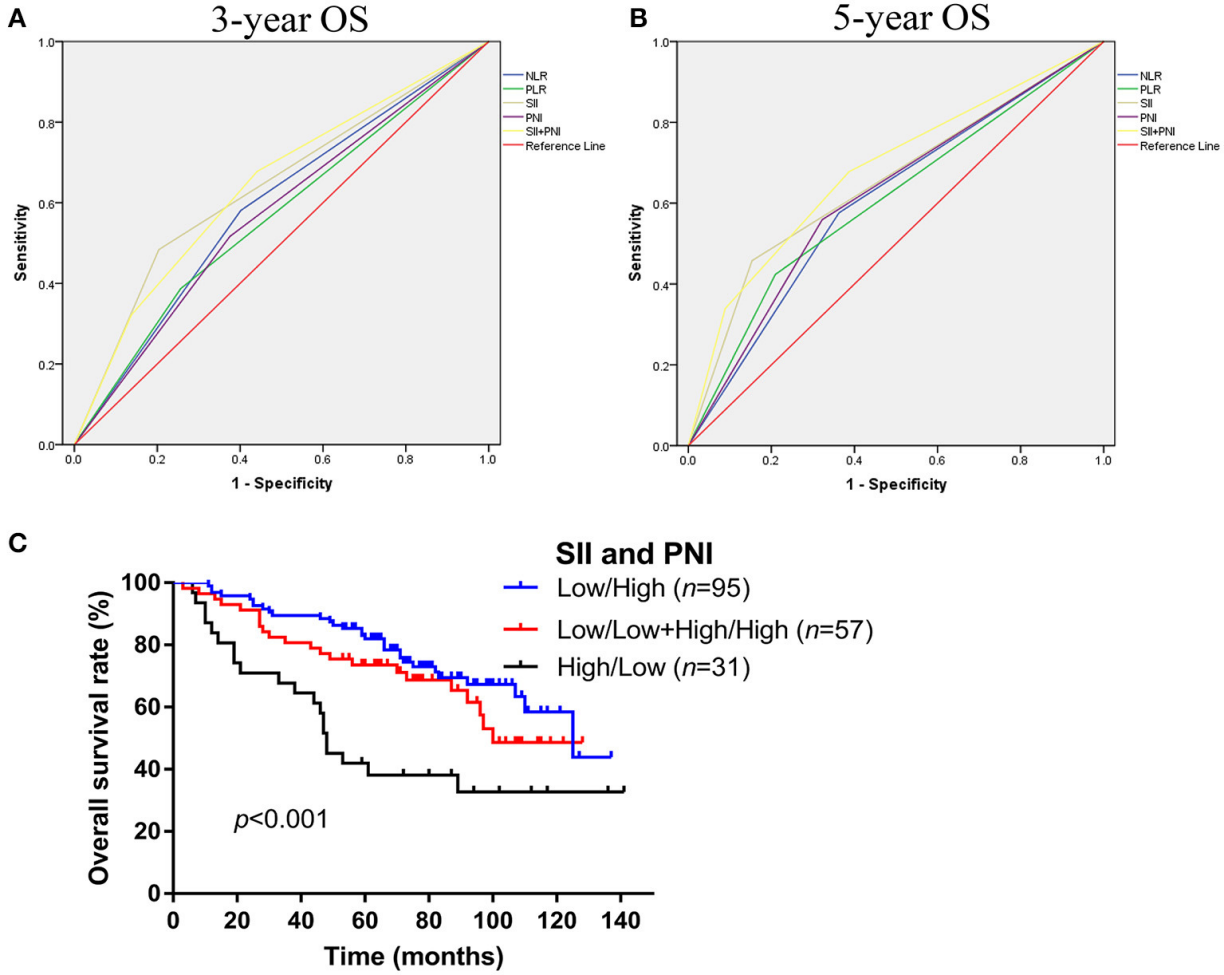
Comparison the predictive values of NLR, PLR, SII, PNI, and SII+PNI for OS in skull base chordoma. **(A)** ROC curves of NLR, PLR, SII, PNI, and SII+PNI for 3-year OS. **(B)** ROC curves of NLR, PLR, SII, PNI, and SII+PNI for 5-year OS. **(C)** Kaplan–Meier survival curves indicated patients with high SII and low PNI had worse OS (*p* < 0.001). NLR, neutrophil lymphocyte ratio; PLR, platelet lymphocyte ratio; SII, systemic immune-inflammation index; PNI, prognostic nutritional index; OS, overall survival; ROC, receiver operating characteristic.

As shown in Figure 2, we further compared the predictive ability of those inflammation associated markers for OS at 3 (Figure 3A) and 5 years (Figure 3B) by ROC curve. The area under the curve (AUC) of NLR, PLR, SII, and PNI for 3-year OS was 0.590, 0.565, 0.640, 0.571, and for 5-year OS, the AUC was 0.607, 0.607, 0.652, and 0.618, respectively (Table 3). Considering that SII and PNI had a greater AUC and were possible independent factors for OS in the multivariable Cox analysis, a combined analysis of SII and PNI (SII + PNI) was performed. We found that patients with SII ≥600.50 and PNI <54.55 had worst OS (median 48 months), compared to other groups using Kaplan-Meier analysis (median OS: 100 months for high SII/high PNI or low SII/high PNI group; 125 months for low SII/high PNI patients, *p* < 0.001) (Figure 3C). Moreover, ROC curve analysis showed SII+PNI had the greatest AUC among the inflammation-based markers (3-year OS = 0.643; 5-year OS = 0.681), indicating SII+PNI was a more accurate predictor of survival among the four inflammation markers and may serve as a readily available grading system for skull base chordoma patients (Figures 3A,B, Table 3).

**Table 3:** Predictive ability of NLR, PLR, SII, PNI, SII+PNI for 3-year and 5-year OS in skull base chordoma.

**Variables**	**3-year OS**			**5-year OS**		
	**AUC**	**95% CI**	***P*-value**	**AUC**	**95% CI**	***P*-value**
NLR	0.590	0.479–0.700	0.116	0.607	0.519–0.695	0.020^*^
PLR	0.565	0.451–0.680	0.253	0.607	0.517–0.697	0.019^*^
SII	0.640	0.526–0.754	0.014^*^	0.652	0.563–0.742	0.001^*^
PNI	0.571	0.459–0.682	0.216	0.618	0.530–0.706	0.010^*^
SII+PNI	0.643	0.533–0.752	0.012^*^	0.681	0.595–0.767	<0.001^*^

* *p < 0.05; NLR, neutrophil lymphocyte ratio; PLR, platelet lymphocyte ratio; SII, systemic immune-inflammation index; PNI, prognostic nutritional index; OS, overall survival; AUC, area under the curve; CI, confidence interval*.

## Discussion

Studies have increasingly evaluated prognostic factors in skull base chordoma. Several clinical features, such as age, gender, extent of resection, pathology type, and tumor progression, have been recognized as potential prognostic indicators, though consensus has not been reached (28, 29). However, those factors were mainly based on the clinicopathologic data and the role of preoperative inflammation markers from blood tests were not fully evaluated in chordoma. In this study, we further characterized the clinical and prognostic value of four inflammatory and nutritional markers and demonstrated that all four markers were associated with OS. Furthermore, SII was an independent prognostic factor, and PNI trended toward significant for OS in the multivariable Cox analysis in skull base chordoma. We additionally compared the predictive accuracy of these markers and demonstrated that the joint effect of SII and PNI showed the best predictive accuracy for 3-year or 5-year survival in skull base chordoma patients. Moreover, patients with high SII (≥600.50) and low PNI (<54.55) had worst outcomes (median OS, 48 months). Our study proposed a potential prognostic grading system for chordoma patients and indicated that patients with high SII and low PNI should receive a more radical therapy strategy and closer monitoring than those who do not have those characteristics. Lastly, consistent with previous studies, our results indicated that degree of resection and tumor recurrence were independent prognostic factors for OS in skull base chordoma. To the best of our knowledge, this is the first study to explore the clinical implications of PNI in relation to other inflammation markers and patients' outcomes in chordoma.

Research has shown that there is a relationship between the inflammatory response, tumor progression and immunity, and the inflammation response, assessed via several inflammatory markers NLR, PLR, and SII, are correlated with survival of patients with tumor (10). In accordance with Hu et al. (21), our retrospective study revealed that NLR, PLR, and SII were all risk factors for OS in skull base chordoma patients and SII was further identified as an independent prognostic factor for OS in skull base chordoma patients. Therefore, better understanding the role of these markers in chordoma will help to facilitate the exploration of the relationship between inflammation, immunity, and chordoma. However, the underlying mechanisms need further evaluation. For example, recent studies have revealed that patients with high NLR, PLR, and/or SII often possess elevated neutrophils, high thrombocytes or decreased lymphocytes, indicating increased inflammation and a decreased immune response. Neutrophils reportedly engage in tumor formation and progression through several mechanisms including vascular endothelial growth factor-mediated angiogenesis and tumor immunological suppression (9, 30). Moreover, thrombocytes, which are bilaterally related to tumor cells, contribute to tumor stromal interaction, angiogenesis and the escape of immune surveillance, leading to further cancer growth and metastases (31). Lymphocytes, including cytotoxic lymphocytes and CD4^+^ T helper cells reflect host immunity status and mediate the host immune response to cancer; furthermore, lymphocytes have antitumor effect via suppression of cancer cell proliferation and invasion and promotion of tumor cell apoptosis (8). Therefore, patients with high SII may benefit from anti-inflammation therapy or immunotherapy, suggesting anti-inflammatory treatment or immunotherapy may be an effective therapeutic strategy for skull base chordoma patients. These studies may help elucidate why patients with high SII have worse outcomes, indicating the potential for anti-inflammatory treatment in skull base chordoma patients, though further investigations are required.

PNI, a well-designed marker composed of albumin and lymphocytes that assesses the immunological and nutritional states in humans, is recognized as an effective negative prognostic factor in cancers (32, 33). However, no previous studies had characterized PNI and its prognostic value in chordoma. This study is the first to have assessed the level of PNI in chordoma, and, in accordance with previous studies in other tumors, our results indicated that the level of PNI was correlated with patients' age, and low PNI (<54.55) patients had significantly poorer OS than those with high PNI, and multivariable Cox analysis further indicated that PNI seemed to be an independent prognostic factor for skull base chordoma patients. More importantly, a combinative analysis of SII and PNI revealed that patients with high SII and low PNI had the lowest survival rates and skull base chordoma patients with low SII and high PNI had the best overall survival. Moreover, combinative analysis of SII and PNI had superior predictive power to other markers for survival in skull base chordoma. The underlying mechanisms of PNI in chordoma remain unclear and we hypothesize that low PNI patients harbor low levels of albumin and potential malnutrition, which may mediate host immune-suppression in coordination with lymphocytopenia (10).

Interestingly, recent studies revealed that tumor immune microenvironment was a promising predictor for outcome in chordoma patients (34). Given the potential relationship between systemic immune-inflammation status and tumor immune microenvironment (35, 36), we will explore the role of tumor-infiltrating immune cells using transcriptomics and immunohistochemistry, and analyze their associations with peripheral blood indexes in our future work.

Some limitations exist in our study. First, this was a retrospective study in a single medical center with relatively small scale, therefore this study may be subject to bias. However, the long patient follow-up time may partly remedy this limitation. Second, the level and prognostic value of C-reactive protein (CRP), a marker of inflammatory response (37, 38), was not evaluated in the present cohort, because CRP is not a routine preoperative blood test index in routine clinical visits. Further studies are warranted to assess the prognostic and predictive value of CRP and its possible combination with SII and PNI. Finally, considering that there are no widely accepted cut points for these inflammatory markers, we determined the cut-off value by OS analysis. Large, multi-site prospective cohort studies are necessary to validate the results.

In conclusion, our results demonstrate that SII and PNI may be effective prognostic indicators of OS for primary skull base chordoma patients after surgical resection. A combinative use of SII and PNI is recommended to increase the prognostic ability for survival in skull base chordoma.

## Data Availability Statement

The original contributions presented in the study are included in the article/Supplementary Material, further inquiries can be directed to the corresponding author.

## Ethics Statement

The studies involving human participants were reviewed and approved by the ethics review board of Beijing Tiantan Hospital. The patients/participants provided their written informed consent to participate in this study.

## Author Contributions

ML and YZhang designed the study. SW, YZhai, SZ, CL, and JD collated the data. ML and JB analyzed the data. ML and YZhang wrote the manuscript. All authors have read and approved the final manuscript.

## Conflict of Interest

The authors declare that the research was conducted in the absence of any commercial or financial relationships that could be construed as a potential conflict of interest.

## Abbreviations

NLR: neutrophil lymphocyte ratio
PLR: platelet lymphocyte ratio
SII: systemic immune-inflammation index
PNI: prognostic nutritional index
OS: overall survival
HR: hazard ratio
CI: confidence interval
ROC: receiver operating characteristic
AUC: area under the curve
CRP: C-reactive protein.
