# Supplementary figures and images for: Clinical Implication of Systemic Immune-Inflammation Index and Prognostic Nutritional Index in Skull Base Chordoma Patients
Source: Front Oncol. 2021 Feb 25;11:548325. doi: 10.3389/fonc.2021.548325 (PMC7947628; doi:10.3389/fonc.2021.548325)

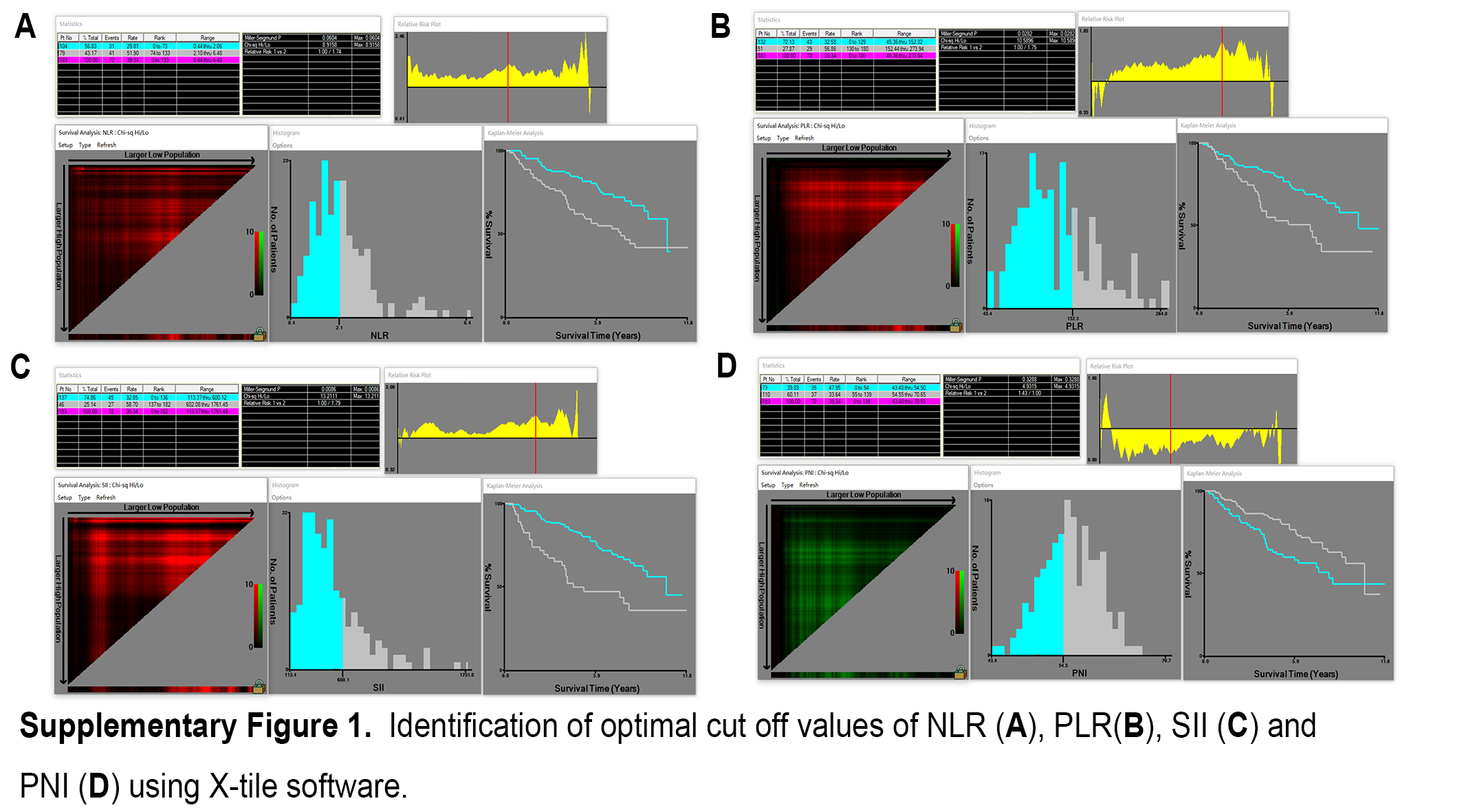

Supplement: Supplementary file 1 [file Image_1.TIF]

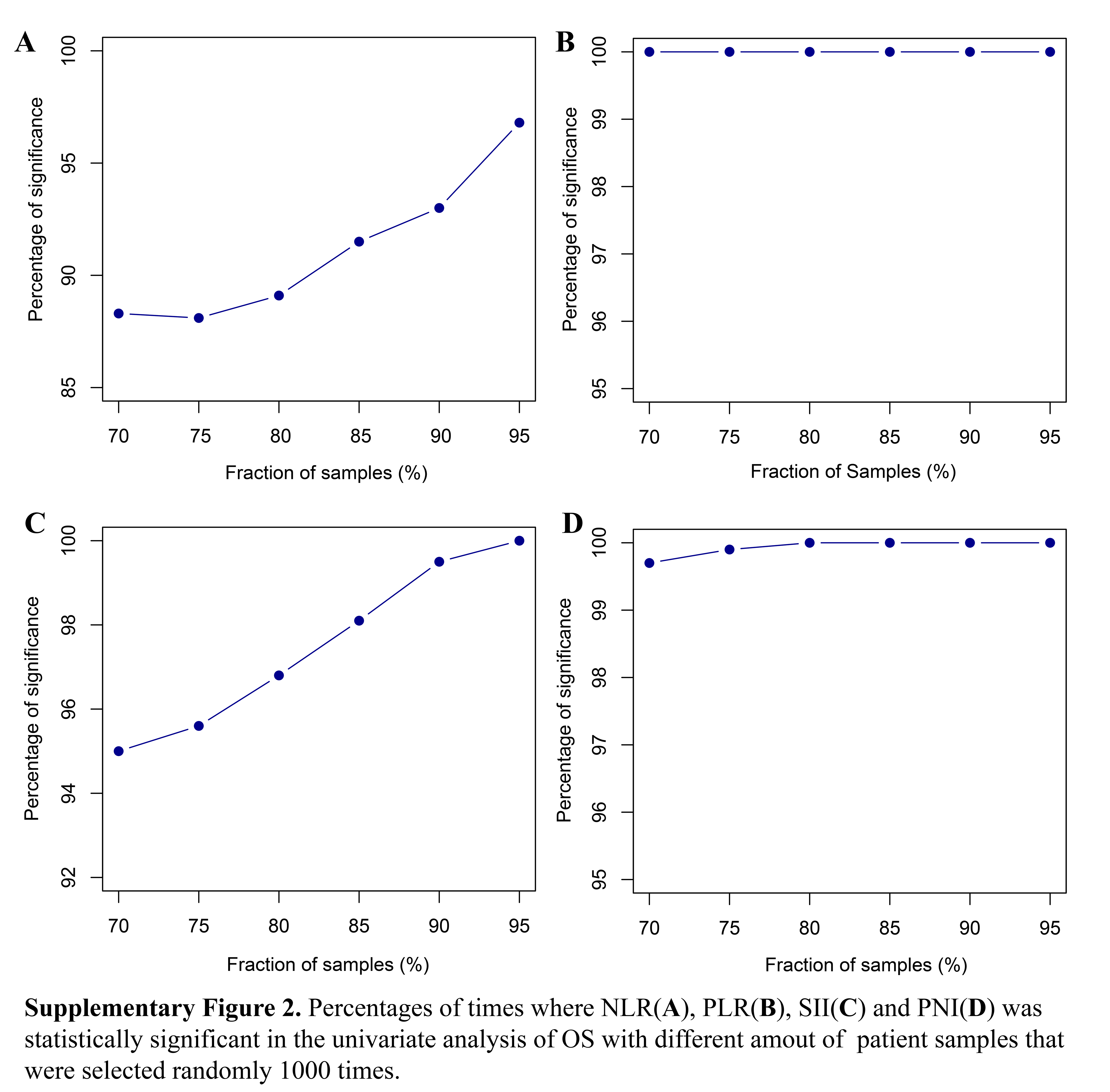

Supplement: Supplementary file 2 [file Image_2.TIF]
